# 2950. Guideline discordant antibiotic treatment in the Emergency Department is associated with increased adverse events and length of stay

**DOI:** 10.1093/ofid/ofad500.189

**Published:** 2023-11-27

**Authors:** Maddy Breeden, Rishi Chanderraj, Lindsay A Petty

**Affiliations:** Michigan Medicine, Ann Arbor, MI; University of Michigan, Ann Arbor, MI; University of Michigan, Ann Arbor, MI

## Abstract

**Background:**

Skin and soft tissue infections (SSTI) are the 3^rd^ most common infectious disease treated in the Emergency Department (ED). Observational studies have shown poor adherence to IDSA guidelines in the ED. While there are numerous descriptive studies of guideline-discordant antibiotic treatment for SSTI in the ED, less is known about the impact of discordance on outcomes.

Figure 1

**Figure:**
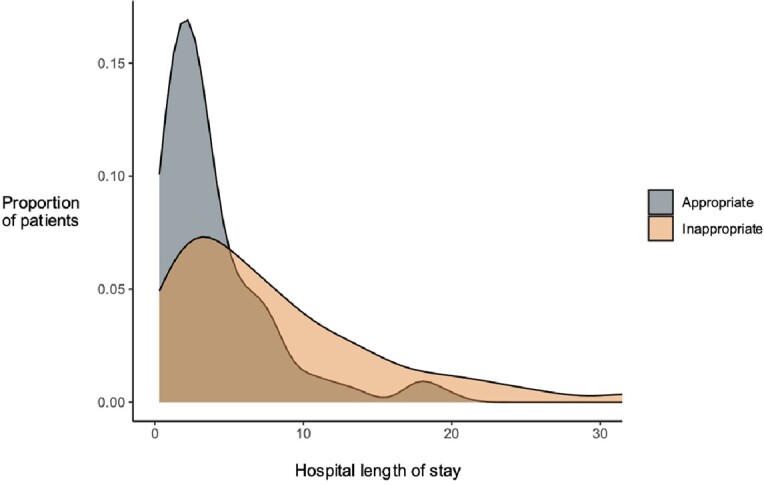

Inappropriate antibiotic treatment is associated with an increased hospital stay in patients admitted with a skin and soft tissue infection (Mean length of stay 4.62 days among appropriately treated patients and 10.63 days in inappropriately treated patients).

**Methods:**

In this retrospective cohort study, 214 consecutive SSTI admissions treated with IV antibiotics at the Ann Arbor Veterans Affairs Hospital from 2020-2022 were reviewed by 2 Infectious Diseases physicians. Guideline concordance was adjudicated based on IDSA guidelines. Outcomes were hospital length of stay (LOS) and incidence of hospital-associated adverse events as defined by the Center for Medicare Services. The primary exposure was the concordance of antibiotic treatment with IDSA guidelines. Covariates included demographics, the burden of medical comorbidities by the Charlson index, and the severity of illness measured by the Severe Organ Failure Score (SOFA). We modeled the risk of hospital-associated adverse events with a multivariable logistic regression model and hospital LOS with a multivariable Poisson model.

**Results:**

Of 214 patients with SSTI treated with IV antibiotics, only 15.7% (N=19) received guideline-concordant therapy, and only 32% (N=69) had indications for IV treatment. 27 patients (12.6%) had a hospital-associated adverse event. Vancomycin was the most common inappropriate antibiotic (n=76, 35%), and kidney injury was the most common adverse event (n=12, 9.8%). Guideline-discordant antibiotic treatment was associated with an increased length of stay after adjusting for covariates (0.83 more hospital days in guideline discordant, 95% CI 0.73-0.93 hospital days, p=0.027, Figure 1), and increased odds of hospital-associated adverse events (OR 1.03, 95% CI 1.002-1.08, p=0.04).

**Conclusion:**

Guideline-discordant therapy for SSTI in the ED was associated with unnecessary IV treatment, longer hospitalization, and increased hospital-associated adverse outcomes. Stewardship interventions should target treatment of SSTI in the ED to improve patient outcomes.

**Disclosures:**

**All Authors**: No reported disclosures

